# The association between estradiol levels and cognitive function in postmenopausal women

**Published:** 2018-07

**Authors:** Soheila Gholizadeh, Shahideh Jahanian Sadatmahalleh, Saeideh Ziaei

**Affiliations:** *Department of Midwifery and Reproductive Health, Faculty of Medical Sciences, Tarbiat Modares University, Tehran, Iran. *

**Keywords:** Cognitive function, Estradiol level, Menopause

## Abstract

**Background:**

Levels of estradiol decreases as women arrive the menopausal transition and enter to a low, steady level during the early postmenopause. In addition, memory dysfunction are highly prevalent during this period.

**Objective:**

Our study was designed to determine whether endogenous levels of estradiol are related to cognitive function in postmenopausal.

**Materials and Methods:**

The cross-sectional study was conducted between November 2015 to February 2016 on 209 healthy postmenopausal women. The women filled out the Montreal Cognitive Assessment (MoCA) scale. Then, estradiol level was tested for association with cognitive function adjusted for factors supposed to confound this association.

**Results:**

The prevalence of cognitive dysfunction; MoCA points ≤26 in our participants was 62.7%, and mean±SD of estradiol level was 14.92±10.24pg/ml in participants with cognitive dysfunction in comparison with 21.67±14.92pg/ml in those with normal cognitive function (p<0.001). There were significant association between MoCA points with estradiol level (p<0.001) and educational status (p<0.001).

**Conclusion:**

Estradiol replacement therapy in postmenopausal women with low endogenous estradiol levels and decreased cognitive function might be necessary.

## Introduction

As women arrive in the menopausal transition and during the early postmenopausal stage circulating levels of estradiol diminishes. Memory dysfunction is highly common during this period (1-3). While this topic remains one of ongoing debate, the basic question of whether endogenous serum of estradiol influence cognitive function remains unknown. "In older postmenopausal women, relatively higher serum estradiol levels have been associated with the better memory and global cognitive function, (4) episodic memory, (5, 6) and executive function; (7) however, there are not consistent (8, 9)." Low estrogen levels associated with Alzheimer’s disease risk in late postmenopause women, (10) however again these findings are controversial (11, 12). Few studies have determined potential links between cognitive function and serum estrogens levels in early postmenopausal women, although this period is the time when women are most likely to use hormone replacement therapy. Moreover, in this age group, studies have not found significant relations between estradiol levels and cognitive function (13, 14).

The objective of the present study was to consider whether endogenous levels of estradiol are associated with cognitive function in postmenopausal women who do not use hormone therapy.

## Materials and methods

The cross-sectional study was conducted on 209 healthy postmenopausal women referred to Arash Hospital, Tehran, Iran, between November 2015 to February 2016. Sample size was calculated using coefficient 99%, power level set at 0.95, and the correlation coefficient set to detect 0.31 Pearson’s r between estradiol levels and total MoCA points. The estimated, minimum sample size was 189 participants. Thus, 209 consecutive postmenopausal women who had inclusion criteria were recruited for the study. Inclusion criteria were: 1) age: 45-65 yr, 2) intact uterus and at least one ovary, 3) menstruation interruption for at least 1 yr and not more than 10 yr, 4) serum FSH concentration over 40 IU/ml, 5) no use of hormone therapy during past six months, 6) no chronic diseases such as cancer, diabetes, hypertension, cardiovascular diseases and psychiatric diseases, 7) and no smoking.

The women filled out the MoCA (Montreal Cognitive Assessment) scale and a questionnaire containing personal data. Venous blood sample were collected in the morning after an overnight fast from the women. Levels of estradiol were estimated using electrochemiluminescence method. Then, estradiol level was tested for association with cognitive function adjusted for factors supposed to confound this association.

The MoCA was created and validated by Nasreddine and colleagues (15). It is one page 30-point test administered in approximately 10 min. The MoCA assesses several cognitive domains. The full-scale point’s range is 0-30; scores ≥26 are considered normal, while scores less than 26 are abnormal and suggestive of developing mild cognitive impairment (MCI). Psychometric properties of this scale have been studied in several studies and its validity and reliability have been confirmed (16).

**Ethical consideration**

The study was approved by the Ethics Committee of Tarbiat Modares University of Medical Sciences (EC# 523850) and all subjects gave written informed consent.

**Statistical analysis**

Estradiol levels and MoCA points were not normally distributed according to K-S test. Therefore nonparametric tests were used. First, the association between MoCA points, women age, age at menarche, age at menopause, age at first pregnancy, age at marriage, BMI and gravity with estradiol level were analyzed using Spearman Correlation test. Second, the association between educational and economic status with MoCA points were evaluated by Kruskal-Wallis test.

On stepwise multiple regression analysis, the correlation between age at first pregnancy, educational status, estradiol level, gravidity, age at marriage as independent factors, and MoCA points as a dependent factor were calculated. A 95% confidence interval (CI) was used to describe the strength of association. All statistical analyses were performed by the SPSS software (Statistical Package for the Social Sciences, version 20.0, SPSS Inc., Chicago, IL, USA). A value of p<0.05 was considered significant.

## Results

The prevalence of cognitive dysfunction; MoCA points ≤26 in our participants was 62.7%, and mean±SD of estradiol level was 14.92±10.24 in participants with cognitive dysfunction in comparison with 21.67±14.92 in those with normal cognitive function. The means of age, age at menarche, age at marriage, age at first pregnancy, age at menopause, gravidity, estradiol levels, MoCA points and also socioeconomic and BMI status are shown in Table I. Table II shows the correlate between MoCA points and dependent variables of interest. MoCA points was positively associated with age at first pregnancy (p<0.001), age at marriage (p<0.001), estradiol level (p<0.001) and was negatively associated with gravidity (p<0.001).

The association between socioeconomic statuses with MoCA points is shown in Table III. High educated participants have more MoCA points (p<0.001). Table IV shows the correlation between estradiol levels and MoCA domains separately. Estradiol level was associated with short memory (p<0.001) attention (p<0.001), and executive domains (p<0.01). For better evaluation of the relationship between MoCA points as a dependent factor and mentioned independent factors, multiple regression analysis was conducted. There were significant association between MoCA points with estradiol level (p<0.01) and educational status (p<0.001) (Table V).

**Table I:** Demographic characteristic of the participants

**Variables**		**N (%) or Mean ± SD**	
Age (yr)*		56.98 ± 4.35	
Age at menarche (yr)*		13.18 ± 1.37	
Age at marriage (yr)*		18.41 ± 5.96	
Age at first pregnancy (yr)*		19.49 ± 6.26	
Age at menopause (yr)*		49.88 ± 3.62	
Gravidity*		4.27 ± 1.90	
BMI*		28.24 ± 4.41	
Estradiol levels (pg/ml)*		17.22 ± 11.07	
MoCA points*		24.41 ± 2.58	
Educational status**			
	Primary school		39 (18.7)
	Second school		130 (62.2)
	University		40 (19.1)
Economic status**			
	Poor		70 (33.5)
	Moderate		96 (45.9)
	good		43 (20.6)

MoCA: Montreal cognitive assessment

BMI: Body mass index

* Data presented as Mean±SD

** Data presented as N (%)

**Table II:** Correlation between MoCA points and interested variables*

**Parameters**	**r**	**p-value**
Age (yr)	-0.091	0.188
Age at menarche (yr)	0.061	0.380
Age at marriage (yr)	0.362	<0.001
Age at first pregnancy (yr)	0.319	<0.001
Age at menopause (yr)	0.026	0.704
Gravidity	-0.235	<0.001
BMI	0.006	0.933
Estradiol level (pg/ml)	0.377	<0.001

BMI: Body mass index

r: Spearman correlation

* Spearman correlation test

**Table III:** The association between socioeconomic status with MoCA points*

		**MoCA points**	**p-value**
Educational status			
	Primary school	21.58 ± 2.19	<0.001
	Secondary school	24.59 ± 2.14	
	University	26.45 ± 1.61	
Economic status			
	Poor	24.14 ± 2.56	0.501
	Moderate	24.47 ± 2.64	
	Good	24.69 ± 2.50	

Data presented as mean±SD.

MoCA: Montreal cognitive assessment

* Kruskal-wallistest

**Table IV:** Correlation between estradiol level and MoCA domains*

**MoCA domains**	**r**	**p-value**	**Mean ± SD**
Visuospatial ability domain	0.127	0.066	3.02 ± 0.77
Short memory domain	0.232	<0.001	2.89 ± 0.79
Attention domain	0.293	<0.001	5.22 ± 1.01
Language domain	0.126	0.070	4.96 ± 0.23
Executive function domain	0.305	<0.001	1.81 ± 1.20
Orientation domain	0.094	0.175	5.93 ± 0.25
Total	0.377	<0.001	24.41 ± 2.58

MoCA: Montreal cognitive assessment

r: Spearman correlation

* Spearman correlation test

**Table V:** Stepwise multiple linear regression model for MoCA points*

	**B**	**SE**	**β**	**p-value**
Constant	20.08	0.407		<0.001
Estradiol level	0.044	0.013	0.187	<0.001
Educational status	1.410	0.144	0.550	<0.001

B: Unstandardized (B) regression coefficients

SE: Standard error

β: Standardized (Beta) regression coefficients.

* MoCA points as dependent variable, and gravidity, age at marriage, age at first pregnancy, estradiol level, and educational status as independent variables. Adjusted R2=0.621, There were no significant relationships between other variables.

## Discussion

Our study, primary, investigate to determine whether endogenous levels of estradiol are related to specific domains of cognitive function in postmenopausal women. In our study, estradiol level was associated with short memory, attention, and executive MoCA domains. There is some evidence from the past 30 yr, presents that estradiol modulates cognitive function in animals and human. "The effect begins in uteri when estrogens guide the sexual differentiation of various brain regions controlling reproduction and some cognitive functions." Estrogen modulations on the nervous system continue through adulthood when gonadal hormone highly secrets. "It has been emphasized that estrogens can act at membrane receptors to activate intracellular signaling mechanisms that change cellular function." Also, some new documents show estrogens are synthesized locally in the brain and therefore rapidly change cognitive functions (17).

Secondary, we evaluated the association of cognitive function with socioeconomic status. Cognitive function positively correlated with age at first pregnancy; age at marriage and educational status, but negatively correlated with gravidity. Our findings are consistent with several studies that reported a positive correlation between cognitive function and educational status, (18, 19) but are inconsistent with those of other studies that reported significant positive correlation with age. (20, 21). Differences in the mean of participants, age and the usage of a variety of psychometric instruments may have contributed to this inconsistent finding. The mean of the participants' age in our study was 56.89±4.53 yr and they were in the early postmenopausal midlife period.

We found significant positive association between cognitive function with age at first pregnancy and age at marriage while it was negatively associated with gravidity. The cross-sectional associational research design limited the data interpretation to the correlational association between these variables. Moreover, these findings cannot be generalized across the postmenopausal community because of different cultural and religious status.

For better evaluation of the relationship between cognitive function as dependent variable and gravidity, age at marriage, age at first pregnancy, estradiol level and educational status as an independent variable, multiple linear regression analysis were conducted. There were associated between global cognitive function with estradiol level and educational status and there was no meaningful relationship between the other mentioned independent variables. Our findings are consistent with studies that reported a significant correlation between estradiol level and cognitive function, (4, 5, 7, 17) but are inconsistent with other studies that reported no relationship between them (14, 22, 23). In general, the relationship between estradiol level and cognitive function have generated very mixed findings. Age and gender of the participants, differences in study approach, insufficient sample size and the usage of a variety of psychometric instruments may explain these discrepancies.

We use MoCA for consideration of cognitive function. It is a brief cognitive screening tool with high specificity and sensitivity (87% and 90%, respectively) for detecting Mild Cognitive Impairment (MCI) as currently conceptualized in patients performing normally on mini-mental state examination (15).

Our study has some strength. This is a population-based study of participated women and analyses adjusted for a number of parameters that could potentially confound the association of cognitive function with hormone level. The results of our study support that postmenopausal women with higher levels of estradiol have less likely to discomfort from cognitive impairment and clinicians should evaluate the possibility of low estradiol level as a helping factor in postmenopausal women with decreased cognitive function.

## Conclusion

The present observations suggest that estradiol replacement in postmenopausal women with low endogenous estradiol levels might be necessary.
